# Post–COVID-19 Symptoms 2 Years After SARS-CoV-2 Infection Among Hospitalized vs Nonhospitalized Patients

**DOI:** 10.1001/jamanetworkopen.2022.42106

**Published:** 2022-11-15

**Authors:** César Fernández-de-las-Peñas, Jorge Rodríguez-Jiménez, Ignacio Cancela-Cilleruelo, Angel Guerrero-Peral, José D. Martín-Guerrero, David García-Azorín, Ana Cornejo-Mazzuchelli, Valentín Hernández-Barrera, Oscar J. Pellicer-Valero

**Affiliations:** 1 Department of Physical Therapy, Occupational Therapy, Physical Medicine and Rehabilitation, Universidad Rey Juan Carlos, Madrid, Spain; 2 Headache Unit, Department of Neurology, Hospital Clínico Universitario de Valladolid, Valladolid, Spain; 3 Department of Medicine, Universidad de Valladolid, Valladolid, Spain; 4 Intelligent Data Analysis Laboratory, Department of Electronic Engineering, ETSE (Engineering School), Universitat de València, Valencia, Spain; 5 Gerencia de Atención Primaria Valladolid Este, Valladolid, Spain; 6 Department of Public Health, Universidad Rey Juan Carlos, Madrid, Spain

## Abstract

**Importance:**

Identification of long-term post–COVID-19 symptoms among hospitalized and nonhospitalized patients is needed.

**Objective:**

To compare the presence of post–COVID-19 symptoms 2 years after acute SARS-CoV-2 infection between hospitalized and nonhospitalized patients.

**Design, Setting, and Participants:**

A cross-sectional cohort study was conducted at 2 urban hospitals and general practitioner centers from March 20 to April 30, 2020, among 360 hospitalized patients and 308 nonhospitalized patients with acute SARS-CoV-2 infection during the first wave of the pandemic. Follow-up was conducted 2 years later.

**Main Outcomes and Measures:**

Participants were scheduled for a telephone interview 2 years after acute infection. The presence of post–COVID-19 symptoms was systematically assessed, with particular attention to symptoms starting after infection. Hospitalization and clinical data were collected from medical records. Between-group comparisons and multivariate logistic regressions were conducted.

**Results:**

A total of 360 hospitalized patients (162 women [45.0%]; mean [SD] age, 60.7 [16.1] years) and 308 nonhospitalized patients (183 women [59.4%]; mean [SD] age, 56.7 [14.7] years) were included. Dyspnea was more prevalent at the onset of illness among hospitalized than among nonhospitalized patients (112 [31.1%] vs 36 [11.7%]; *P* < .001), whereas anosmia was more prevalent among nonhospitalized than among hospitalized patients (66 [21.4%] vs 36 [10.0%]; *P* = .003). Hospitalized patients were assessed at a mean (SD) of 23.8 (0.6) months after hospital discharge, and nonhospitalized patients were assessed at a mean (SD) of 23.4 (0.7) months after the onset of symptoms. The number of patients who exhibited at least 1 post–COVID-19 symptom 2 years after infection was 215 (59.7%) among hospitalized patients and 208 (67.5%) among nonhospitalized patients (*P* = .01). Among hospitalized and nonhospitalized patients, fatigue (161 [44.7%] vs 147 [47.7%]), pain (129 [35.8%] vs 92 [29.9%]), and memory loss (72 [20.0%] vs 49 [15.9%]) were the most prevalent post–COVID-19 symptoms 2 years after SARS-CoV-2 infection. No significant differences in post–COVID-19 symptoms were observed between hospitalized and nonhospitalized patients. The number of preexisting medical comorbidities was associated with post–COVID-19 fatigue (odds ratio [OR], 1.93; 95% CI, 1.09-3.42; *P* = .02) and dyspnea (OR, 1.91; 95% CI, 1.04-3.48; *P* = .03) among hospitalized patients. The number of preexisting medical comorbidities (OR, 3.75; 95% CI, 1.67-8.42; *P* = .001) and the number of symptoms at the onset of illness (OR, 3.84; 95% CI, 1.33-11.05; *P* = .01) were associated with post–COVID-19 fatigue among nonhospitalized patients.

**Conclusions and Relevance:**

This cross-sectional study suggested the presence of at least 1 post–COVID-19 symptom in 59.7% of hospitalized patients and 67.5% of nonhospitalized patients 2 years after infection. Small differences in symptoms at onset of COVID-19 were identified between hospitalized and nonhospitalized patients. Post–COVID-19 symptoms were similar between hospitalized and nonhospitalized patients; however, lack of inclusion of uninfected controls limits the ability to assess the association of SARS-CoV-2 infection with overall and specific post–COVID-19 symptoms 2 years after acute infection. Future studies should include uninfected control populations.

## Key Points

**Question:**

What is the prevalence of post–COVID-19 symptoms among hospitalized and nonhospitalized patients 2 years after acute infection?

**Findings:**

This cross-sectional study found that the proportion of patients with at least 1 post–COVID-19 symptom 2 years after acute infection was 59.7% for hospitalized patients and 67.5% for those not requiring hospitalization. No significant differences in post–COVID-19 symptoms were seen between hospitalized and nonhospitalized patients.

**Meaning:**

Similar rates of post–COVID-19 symptoms between hospitalized and nonhospitalized patients suggest that, among all patients who contract COVID-19, these sequelae deserve attention.

## Teaser

This cross-sectional study compares the presence of post–COVID-19 symptoms 2 years after acute SARS-CoV-2 infection between hospitalized and nonhospitalized patients.

## Introduction

SARS-CoV-2, the virus causing COVID-19, has changed the world in the last 2 years. After the worldwide outbreak leading to millions of acute cases and thousands of deaths, another important development is the occurrence or persistence of symptoms after the acute phase of SARS-CoV-2 infection (ie, long COVID^1^ or post–COVID-19).^2^ More than 100 post–COVID-19 symptoms affecting multiple systems (eg, cardiovascular, neurologic, respiratory, and musculoskeletal) have been described.^3^ Evidence supports that individuals exhibiting post–COVID-19 symptoms report worse health-related quality of life.^4^ Several reviews and meta-analyses investigating the prevalence of post–COVID-19 symptoms have been published; however, most of them pooled data from both hospitalized and nonhospitalized patients and the follow-up periods were shorter than 6 months.^5,6,7^ It has been suggested that the prevalence of post–COVID-19 symptoms could be different between hospitalized and nonhospitalized patients.^8^ This hypothesis is supported by a recent meta-analysis reporting a pooled prevalence of post–COVID-19 symptoms of 54% (95% CI, 44%-63%) among hospitalized patients and of 34% (95% CI, 25%-46%) among nonhospitalized patients.^9^ In addition, the meta-analysis also observed that the prevalence rates of post–COVID-19 symptoms were different depending on the follow-up period, but data were also available for periods shorter than 6 months. Similar prevalence rates of post–COVID-19 symptoms among nonhospitalized patients were reported by the only meta-analysis investigating post–COVID-19 symptoms among outpatients.^10^

More than 2 years after the the onset of the pandemic and with increasing evidence, meta-analyses have included studies with follow-up periods up to 1 year after acute infection.^11,12^ Again, these meta-analyses pooled data from both hospitalized and nonhospitalized COVID-19 patients.^11,12^ Direct comparison between hospitalized and nonhospitalized patients is scarce because most studies focused on patients requiring hospitalization.^5,6,7,8,9,10,11,12^ van Kessel et al^10^ identified 5 studies directly comparing post–COVID-19 symptoms between hospitalized and nonhospitalized individuals, with follow-up periods of 3 months after infection. Recent studies directly comparing hospitalized vs nonhospitalized patients have included follow-up periods up to 6 months after acute infection.^13,14,15^ Data for follow-up periods longer than 1 year after SARS-CoV-2 infection are still lacking. Accordingly, the main objective of this study was to compare the presence of post–COVID-19 symptoms among hospitalized and nonhospitalized patients at a follow-up period of 2 years. A secondary aim was to identify potential risk factors associated with the development of post–COVID-19 symptoms 2 years after acute infection among hospitalized and nonhospitalized COVID-19 patients.

## Methods

### Participants

This cross-sectional study followed the Strengthening the Reporting of Observational Studies in Epidemiology (STROBE) reporting guideline.^16^ The study included a group of individuals hospitalized due to SARS-CoV-2 infection from 2 urban hospitals and a group of patients infected with SARS-CoV-2 not needing hospitalization who were managed in an outpatient setting by their general practitioners in Spain. All hospitalized and nonhospitalized patients with COVID-19 who were managed at all of the participating centers during the first wave of the pandemic were included in an anonymous database. A random selection of 400 hospitalized and nonhospitalized patients was performed with a randomization software (Excel; Microsoft Corp). Participants had to be infected during the first wave of the pandemic (March 20-April 30, 2020) and not reinfected with subsequent variants. Diagnosis of SARS-CoV-2 infection should have been confirmed at hospital admission or at a general practitioner center with real-time reverse transcription–polymerase chain reaction assay of nasopharyngeal or oral swab samples. The study was approved by the ethics committees of Universidad Rey Juan Carlos, Hospital Universitario Infanta Leonor, and Hospital Universitario Fundación Alcorcón, and it was conducted according to the Helsinki Declaration.^17^ All participants provided verbal informed consent before collecting any data.

### Procedure

Demographic (age, sex, height, and weight), clinical (COVID-19–associated symptoms at onset and preexisting medical comorbidities), and hospitalization (intensive care unit admission and duration of hospital stay) data were collected from medical records. Patients who agreed to participate were scheduled for a telephone interview by trained researchers at a follow-up 2 years after the acute infection. Participants were systematically asked about the presence of symptoms appearing either after hospitalization or after the infection and whether these symptoms persisted at the time of the study. To classify any symptom as COVID-19 related, it needed to be attributable to the infection, not better explained by another underlying medical disorder, and with an onset no later than 1 month after SARS-CoV-2 infection.

The following post–COVID-19 symptoms were systematically assessed: dyspnea, fatigue, anosmia, ageusia, hair loss, pain symptoms, diarrhea, skin rashes, palpitations, brain fog, visual disorders, cough, and loss of concentration.^5,6,7^ However, participants were free to report any symptom that they experienced and considered relevant.

In addition, the Hospital Anxiety and Depression Scale (HADS) was used for evaluating anxiety or depressive symptoms, and the Pittsburgh Sleep Quality Index (PSQI) was used for evaluating sleep quality because both can be properly assessed by telephone.^18^ Both the HADS anxiety (HADS-A; 7 items; range, 0-21 points) and HADS depressive (HADS-D; 7 items; range, 0-21 points) scales were used.^19^ A cutoff score of 12 points or more for the HADS-A was indicative of anxiety symptoms, and a cutoff score of 10 points or more for the HADS-D was indicative of depressive symptoms.^20^ The PSQI (range, 0-21 points) was used to assess sleep quality during the previous month, in which a cutoff of 8.0 points or more was considered indicative of poor sleep quality.^21^

### Statistical Analysis

Data are presented as mean (SD) values or number of cases (percentage), as appropriate. For the main outcome, we compared the differences in post–COVID-19 symptoms among hospitalized and nonhospitalized patients with the χ^2^ test or 1-way analysis of variance tests as needed. The level of significance was set a priori at *P* < .05, with *P* values from all tests being corrected by means of the Holm-Bonferroni correction. For the second outcome, multivariate logistic regressions were conducted to identify the potential association of post–COVID-19 symptoms with variables collected at the acute phase of the infection as the covariates in hospitalized and nonhospitalized patients with COVID-19 separately. Adjusted odds ratios (ORs) with 95% CIs were calculated. Data were analyzed with Stata, version 16.1 (StataCorp LLC) and processed using Python’s library pandas, version 0.25.3.^22^ SciPy, version 1.5.2^23^ was used for conducting the statistical tests, and statsmodels, version 0.11.0^24^ was used for performing *P* value correction.

## Results

From 400 hospitalized patients and 400 nonhospitalized patients randomly selected and invited to participate, 360 hospitalized patients (162 women [45.0%]; mean [SD] age, 60.7 [16.1] years) and 308 nonhospitalized patients (183 women [59.4%]; mean [SD] age, 56.7 [14.7] years) participated (Table 1). Hospitalized patients were older than nonhospitalized patients (mean [SD], 60.7 [16.1] vs 56.7 [14.7] years; *P* = .03) and had a higher mean (SD) weight (77.9 [16.9] vs 72.6 [12.6] kg; *P* < .001). In addition, the proportion of men was higher among hospitalized than nonhospitalized patients (198 [55.0%] vs 125 [40.6%]; *P* = .009).

**Table 1.** Clinical and COVID-19–Associated Onset Data on Hospitalized and Nonhospitalized Patients

Characteristic	Patients, No. (%)		*P* value
	Hospitalized (n = 360)	Nonhospitalized (n = 308)	
Age, mean (SD), y^a^	60.7 (16.1)	56.7 (14.7)	.03
Weight, mean (SD), kg^a^	77.9 (16.9)	72.6 (12.6)	<.001
Height, mean (SD), cm	165.6 (9.6)	164.7 (6.1)	.17
Female^a^	162 (45.0)	183 (59.4)	.009
No. of preexisting comorbidities, mean (SD)	1.0 (1.0)	0.8 (1.0)	.60
Obesity (preexisting)	28 (7.8)	31 (10.1)	.32
Hypertension (preexisting)	120 (33.3)	76 (24.7)	.54
Diabetes (preexisting)^a^	49 (13.6)	15 (4.9)	.01
Asthma (preexisting)	31 (8.6)	18 (5.8)	.17
COPD (preexisting)	15 (4.2)	7 (2.3)	.18
Cardiac disease (preexisting)	43 (11.9)	34 (11.0)	.73
Rheumatologic disease (preexisting)	5 (1.4)	13 (4.2)	.97
No. of COVID-19 symptoms at hospital admission, mean (SD)	2.3 (0.9)	2.5 (1.0)	.09
Fever (COVID-19 onset)	232 (64.4)	169 (54.9)	.45
Dyspnea (COVID-19 onset)^a^	112 (31.1)	36 (11.7)	<.001
Myalgias (COVID-19 onset)	113 (31.4)	94 (30.5)	.81
Cough (COVID-19 onset)	97 (26.9)	69 (22.4)	.17
Headache (COVID-19 onset)	85 (23.6)	87 (28.2)	.17
Diarrhea (COVID-19 onset)	56 (15.6)	32 (10.4)	.06
Anosmia (COVID-19 onset)^a^	36 (10.0)	66 (21.4)	.003
Ageusia (COVID-19 onset)	26 (7.2)	30 (9.7)	.24
Throat pain (COVID-19 onset)	21 (5.8)	34 (11.0)	.58
Vomiting (COVID-19 onset)	11 (3.1)	9 (2.9)	.92
Dizziness (COVID-19 onset)	23 (6.4)	12 (3.9)	.15
Hospital, mean (SD), d	13.0 (13.2)	NA	NA
ICU admission	17 (4.7)	NA	NA

Abbreviations: COPD, chronic obstructive pulmonary disease; ICU, intensive care unit; NA, not applicable.

^a^ Significant at *P* < .05.

Table 1 summarizes clinical and hospitalization data for both hospitalized and nonhospitalized patients with COVID-19. The most frequent symptoms that presented during the acute phase of SARS-CoV-2 infection were fever, dyspnea, myalgia, and cough. Dyspnea was significantly more prevalent at the onset of illness among hospitalized than nonhospitalized patients (112 [31.1%] vs 36 [11.7%]; *P* < .001), whereas anosmia was more prevalent at onset among nonhospitalized patients than hospitalized patients (66 of 308 [21.4%] vs 36 of 360 [10.0%]; *P* = .003). In addition, a greater proportion of hospitalized than nonhospitalized patients had preexisting comorbid diabetes (49 [13.6%] vs 15 [4.9%]; *P* = .01). No other significant differences were identified.

Hospitalized participants were assessed at a mean (SD) of 23.8 (0.6) months after hospital discharge, whereas nonhospitalized patients were assessed at a mean (SD) of 23.4 (0.7) months after the onset of symptoms. The number of patients who exhibited at least 1 post–COVID-19 symptom 2 years after the acute infection was 215 (59.7%) among hospitalized patients and 208 (67.5%) among nonhospitalized patients (*P* = .01). Table 2 shows post–COVID-19 symptoms for both hospitalized and nonhospitalized patients at 2 years after SARS-CoV-2 infection. Fatigue (161 [44.7%] vs 147 [47.7%]), pain (129 [35.8%] vs 92 [29.9%]), and memory loss (72 [20.0%] vs 49 [15.9%]) were the most prevalent post–COVID-19 symptoms 2 years after SARS-CoV-2 infection. No significant differences in post–COVID-19 symptoms were found between hospitalized and nonhospitalized patients. Nonhospitalized patients showed higher levels of anxiety than hospitalized patients (mean [SD] HADS-A score, 1.8 [2.6] vs 1.2 [1.9]; *P* = .04), but the differences were small.

**Table 2.** Post–COVID-19 Symptoms and Psychological Aspects in Hospitalized and Nonhospitalized COVID-19 Survivors

Symptom	Patients, No. (%)		*P* value
	Hospitalized (n = 360)	Nonhospitalized (n = 308)	
No. of post–COVID-19 symptoms, mean (SD)	1.3 (1.4)	1.6 (1.4)	.54
Fatigue	161 (44.7)	147 (47.7)	.44
Dyspnea at rest	14 (3.9)	12 (3.9)	.99
Pain symptoms (including headache)	129 (35.8)	92 (29.9)	.10
Memory loss	72 (20.0)	49 (15.9)	.17
Cognitive blurring or brain fog	18 (5.0)	27 (8.8)	.06
Concentration loss	6 (1.7)	18 (5.8)	.30
Hair loss	27 (7.5)	30 (9.7)	.32
Palpitations or tachycardia	2 (0.6)	6 (1.9)	.12
Rashes	7 (1.9)	9 (2.9)	.41
Gastrointestinal problems	8 (2.2)	14 (4.5)	.10
Diarrhea	0	6 (1.9)	.33
Voice problems	1 (0.3)	5 (1.6)	.10
Ageusia	4 (1.1)	6 (1.9)	.38
Anosmia	16 (4.4)	13 (4.2)	.90
Ocular problems	14 (3.9)	17 (5.5)	.32
Throat pain	6 (1.7)	11 (3.6)	.13
HADS-A score (range, 0-21), mean (SD)^a^	1.2 (1.9)	1.8 (2.6)	.04
HADS-D score (range, 0-21), mean (SD)	1.7 (2.4)	1.8 (2.5)	.42
PSQI score (range, 0-21), mean (SD)	6.5 (3.7)	6.4 (3.5)	.60

Abbreviations: HADS-A, Hospital Anxiety and Depression Scale–anxiety subscale; HADS-D, Hospital Anxiety and Depression Scale–depression subscale; PSQI, Pittsburgh Sleep Quality Index.

^a^ Significant at *P* < .05.

For hospitalized patients, multivariate analysis revealed that, after adjusting for all variables collected at hospitalization, the number of preexisting comorbidities was associated with the presence of post–COVID-19 fatigue (OR, 1.93; 95% CI, 1.09-3.42; *P* = .02) and dyspnea (OR, 1.91; 95% CI, 1.04-3.48; *P* = .03). For nonhospitalized patients, multivariate analysis revealed that, after adjusting for all variables collected at the acute phase of the infection, the number of preexisting medical comorbidities (OR, 3.75; 95% CI, 1.67-8.42; *P* = .001) and the number of symptoms at onset (OR, 3.84; 95% CI, 1.33-11.05; *P* = .01) were associated with the presence of fatigue.

## Discussion

To our knowledge, this is the first study comparing the presence of post–COVID-19 symptoms between hospitalized and nonhospitalized patients 2 years after the infection due to the Wuhan variant. Overall, small differences in COVID-19 onset symptoms without differences in post–COVID-19 symptoms were found among hospitalized and nonhospitalized COVID-19 survivors, reinforcing the hypothesis that post–COVID-19 symptoms are not correlated only with COVID-19 severity.

We observed small differences in COVID-19–associated symptoms at onset between hospitalized and nonhospitalized patients. Dyspnea was more prevalent at onset among hospitalized patients, whereas anosmia was more prevalent at onset among nonhospitalized patients. This finding seems to be expected because dyspnea represents one of the most bothersome symptoms of COVID-19 and is perceived by the patient when the disease is more severe. Anosmia and also ageusia have been used as useful tools for clinical triage of COVID-19 against other respiratory infections during the outbreak,^25^ although they are considered nonbothersome symptoms. These differences could be explained by the fact that individuals experiencing less bothersome and less severe symptoms (eg, anosmia, ageusia, and throat pain) did not seek hospitalization during the first wave of the pandemic.

This study suggested the presence of at least 1 post–COVID-19 symptom in 59.7% of hospitalized patients and 67.5% of nonhospitalized patients 2 years after SARS-CoV-2 infection. This study is the largest follow-up comparison between these populations. Our prevalence rate of post–COVID-19 symptoms among hospitalized patients is similar to the prevalence rates provided in previous meta-analyses^5,6,7,8^; however, our study included the longest follow-up period to date. There is only 1 study including a 2-year follow-up for previously hospitalized patients with COVID-19.^26^ Our results agree with those of Huang et al,^26^ showing that 55% of hospitalized patients exhibited post–COVID-19 symptoms 2 years after hospital discharge. Current data would suggest that a large proportion of hospitalized patients with COVID-19 will exhibit symptoms 2 years after the infection, although further studies are clearly needed.

Data on nonhospitalized patients are based on follow-up periods no longer than 6 months^9,10,13,14,15^; accordingly, we cannot directly compare our results with previous data. Previous studies have reported lower prevalence rates of post–COVID-19 symptoms among nonhospitalized than hospitalized patients.^9,10,13,14,15^ Our results revealed similar proportions of hospitalized and nonhospitalized patients with post–COVID-19 symptoms 2 years after the acute infection, suggesting that, despite having not been hospitalized during the acute phase, the symptoms of long COVID are also found in the nonhospitalized cohort. This finding could be explained by the fact that COVID-19 severity is not a risk factor for the development of long COVID symptoms.^27^

Previous and current data support that fatigue and musculoskeletal pain are highly prevalent symptoms throughout the first years after SARS-CoV-2 infection. The presence of fatigue and dyspnea is associated with a higher post–COVID-19 burden.^28^ However, the prevalence of post–COVID-19 dyspnea in our sample of hospitalized and nonhospitalized patients 2 year after the infection was small. Although dyspnea has been reported as a prevalent post–COVID-19 symptom in previous studies,^9,10^ an analysis of the exponential recovery curve revealed that dyspnea decreased in the years after SARS-CoV-2 infection,^29^ supporting the lower prevalence rates seen in our study. On the contrary, fatigue did not decrease in the same way as dyspnea,^29^ which could explain the high prevalence of fatigue 2 years after COVID-19. These findings support theories suggesting that fatigue probably represents the most prevalent and the most long-lasting post–COVID-19 symptom. Current hypotheses propose that post–COVID-19 fatigue shares common features with myalgic encephalomyelitis or chronic fatigue syndrome.^30^ Similar endothelial dysfunction has been identified among individuals with long COVID and those with myalgic encephalomyelitis or chronic fatigue syndrome.^31^

The identification of risk factors for identifying who might develop long COVID, how long the symptoms last, and whether COVID-19 prompts the presentation of other chronic diseases is crucial for developing treatment strategies. This information on nonhospitalized patients is scarce, to our knowledge. Estiri et al^32^ identified some phenotypes among nonhospitalized patients with COVID-19, but their results were based on a follow-up period shorter than 6 months after the infection. It has been found that female sex and the number of onset symptoms at hospital admission, but not COVID-19 severity, are potential risk factors for long COVID.^27^ We were unable to identify these risk factors in our sample of hospitalized patients because solely the number of previous medical comorbidities was the only variable associated with post–COVID-19 fatigue and dyspnea. We also identified that the number of symptoms in the acute phase of the infection was a risk factor for post–COVID-19 fatigue among nonhospitalized patients, in agreement with previous findings.^27^ This result would agree with the theory that a higher viral load in the acute phase of the infection is associated with an exaggerated immune response, which in turn is associated with the development of long COVID.^33^ A recent study has identified 4 different risk factors associated with SARS-CoV-2 acute infection (ie, presence of type 2 diabetes, SARS-CoV-2 RNAemia, Epstein-Barr virus viremia, and specific autoantibodies) that were associated with the development of long COVID symptoms.^34^

We should not exclude the emotional and social factors surrounding the COVID-19 outbreak from our results. For instance, several COVID-19 outbreak–associated factors (eg, social alarm, somatization, posttraumatic stress disorder, and fear or uncertainty about prognosis) can also play a role in the development of long COVID. However, we observed that our sample of hospitalized and nonhospitalized patients with COVID-19 did not exhibit symptoms of anxiety and depression because their mean HADS-A and HADS-D scores were distant from the established cutoff values.^20^ The fact that individuals with long COVID exhibit anxiety and depressive levels is clear in the literature^35^; however, most studies included shorter follow-up periods. As with fatigue and dyspnea, it is expected that anxiety and depressive levels will decrease during the months or years after the infection.^36^

### Limitations

Although this is the first study comparing post–COVID-19 symptoms between hospitalized and nonhospitalized patients 2 years after the infection, current data should be considered according to their limitations. First, our results can only be applicable to patients with COVID-19 infected with the Wuhan variant and not reinfected. In fact, current data about post–COVID-19 symptoms and SARS-CoV-2 variants of concern suggest that the Alpha and Delta variants overall exhibit less severe post–COVID-19 symptoms than the Wuhan variant^37^ and that the Omicron variant also had less prolonged symptoms than the Delta variant.^38^ In addition, we did not control for vaccination, but most patients reported that they received 2 doses of vaccine without experiencing a significant change in their symptoms. This finding agrees with a recent systematic review reporting that the association of vaccines with outcomes among individuals with existing post–COVID-19 symptoms is still controversial because some studies observed improvement in the symptoms, whereas others did not observe any change or even a worsening of the symptoms.^39^ Second, we did not collect laboratory biomarkers at the acute phase, which could help to elucidate if they are associated with long-term post–COVID-19 symptoms. Third, we collected data through telephonic interviews, a procedure with a potential recall bias but one used extensively in post–COVID-19 research.^9,10^ In addition, post–COVID-19 symptoms were self-reported by patients. It is possible that the use of scales evaluating different symptoms (eg, fatigue or dyspnea) could reveal potential differences between groups. Fourth, the cross-sectional design of the study does not allow for assessment of the evolution of post–COVID-19 symptoms from the acute phase of the infection, making it difficult to exclusively attribute the presence of symptoms 2 years later to SARS-CoV-2. Lack of inclusion of uninfected controls limits the ability to evaluate a direct association of SARS-CoV-2 infection with overall and specific post–COVID-19 symptoms 2 years later. Accordingly, future studies could include uninfected control populations. Fifth, although this study included a longer follow-up period than many other published articles, population-based studies including large samples and addressing the limitations discussed in the study are need to confirm or refute current results.

## Conclusions

This cross-sectional study identified some differences in COVID-19–associated symptoms at onset between hospitalized and nonhospitalized patients. Dyspnea was more prevalent among hospitalized patients, whereas anosmia was more prevalent among nonhospitalized patients. Post–COVID-19 symptoms were similar between hospitalized and nonhospitalized patients; however, the lack of inclusion of uninfected controls limits the ability to evaluate the association of SARS-CoV-2 infection with overall and specific post–COVID-19 symptoms 2 years after the infection. Future studies should include uninfected control populations. Current evidence supports that long COVID will require specific management attention independently of whether the patient has been hospitalized or not.
